# Supplementary material for: Content-rich biological network constructed by mining PubMed abstracts
Source: BMC Bioinformatics. 2004 Oct 8;5:147. doi: 10.1186/1471-2105-5-147 (PMC528731; doi:10.1186/1471-2105-5-147)
Supplement: Additional File 2 — The original results of the above study (non-essential files are deleted to keep the file size under the limit set by BMC bioinformatics). [file 1471-2105-5-147-S2.bz2 › chilibotAdditionalFile2/dip40/92ID8950973E399/html/GRB2_CBL.html]

 


 **GRB2** and **CBL** 
  
Found 100 abstracts in PubMed, retrieved 40.  
 

 What does Google say? 
 PDF only 
| .edu only 

---

**Interactive relationship** (e.g. stimulation, inhibition, etc)

**Stimulatory relationship**- **Cbl**  b constitutively binds  **Grb2**  and becomes associated with Crk L upon TCR stimulation.  Ref: 10022120 Oncogene, 1999
- CD2 crosslinking markedly induced tyrosine phosphorylation of  **Cbl**  associated with  **Grb2**  or CrkL, Shc and LAT, compared with IL 2 stimulation.  Ref: 11792427 Mol Immunol, 2002
- We demonstrate in vivo, that  **cbl**  b, like c  **cbl** , is phosphorylated and recruited to the EGFR upon EGF stimulation and both  **cbl**  proteins can bind to the  **Grb2**  adaptor protein.  Ref: 10086340 Oncogene, 1999
- Here, we demonstrate that  **Grb2**  bound to tyrosine phosphorylated FRS2 alpha forms a ternary complex with  **Cbl**  by means of its Src homology 3 domains resulting in the ubiquitination of fibroblast growth factor FGF receptor and FRS2 alpha in response to FGF stimulation.  Ref: 11997436 Proc Natl Acad Sci U S A, 2002
- SLP 76, a recently cloned  **Grb2**  associated protein, was strongly tyrosine phosphorylated after Fc gammaRI stimulation and was associated with both  **Cbl**  and  **Grb2** .  Ref: 10204582 J Leukoc Biol, 1999
**Neutral relationship**- In vitro binding studies using GST fusion proteins revealed that interactions of  **Grb2**  Shc and CrkL  **Cbl**  were mediated through each SH2 domain in tyrosine phosphorylation dependent manner.  Ref: 11792427 Mol Immunol, 2002
- These results indicate that TCDD modulates the molecular interaction of adaptor complex proteins including Shc,  **Grb2** , Sos, and  **Cbl** .  Ref: 9918770 Biochem Biophys Res Commun, 1998
- ...  **Grb2**  Sos,  **Grb2**  Shc, and  **Cbl**  Shc interactions are highly inducible.  Ref: 10570290 J Immunol, 1999
- SETA is a multifunctional adapter protein with three SH3 domains that binds  **Grb2** ,  **Cbl** , and the novel SB1 proteins.  Ref: 11152963 Cell Signal, 2000
- Evidence that SETA binds to the CD2 protein, the proto oncogene c  **Cbl**   [ **CBL** ] , and the signal transduction molecule  **Grb2** , and can dimerize via its C terminal coiled coil CC domain is also presented.  Ref: 11152963 Cell Signal, 2000
- Interaction of some Ruk isoforms with several signalling proteins, including the p85 regulatory subunit of the Class IA PI 3 kinase, c  **Cbl**  and  **Grb2** , has been demonstrated.  Ref: 12242006 Gene, 2002
- Localization of  **Cbl**  yellow fluorescent protein to these endocytic organelles was dependent on a proline rich domain of c  **Cbl**  that interacts with  **Grb2**  as shown by fluorescence resonance energy transfer microscopy.  Ref: 12839496 Traffic, 2003
- L selectin tyrosine phosphorylates  **cbl**  and induces association of tyrosine phosphorylated  **cbl**  with crkl and  **grb2** .  Ref: 11263968 Biochem Biophys Res Commun, 2001
- These data suggest that the binding of  **Cbl**  to epidermal growth factor receptor through  **Grb2**  is necessary and sufficient for  **Cbl**  function during clathrin mediated endocytosis.  Ref: 12839496 Traffic, 2003
- Immunodepletion studies reveal that  **Grb2**  associated tyrosine phosphorylated p120 kDa protein is  **Cbl** .  Ref: 11399323 Mol Immunol, 2000
- **Grb2**  and Shc are also degraded in an EGF induced  **Cbl**  b dependent fashion.  Ref: 11375397 J Biol Chem, 2001
- Cross linking of FcgammaRI induces the tyrosine phosphorylation of  **Cbl** , which forms a complex with  **Grb2**  and Shc via the  **Cbl**  C terminus.  Ref: 10570290 J Immunol, 1999
- In vitro binding studies using GST fusion proteins demonstrate that  **Cbl**  constitutively associates with the SH3 domains of  **Grb2** , with a preference for the amino terminal domain.  Ref: 11399323 Mol Immunol, 2000
- Thus, in addition to its well characterized role in mediating positive signals,  **Grb2**  can terminate signal transduction by accelerating c  **Cbl**  dependent sorting of active tyrosine kinases to destruction.  Ref: 11823423 EMBO J, 2002
- Although Ron and c  **Cbl**  are found also in physical complexes that include  **Grb2** , these associations are insufficient for productive ubiquitylation of Ron.  Ref: 12802274 Oncogene, 2003
- **Cbl**   **Grb2**  and  **Cbl**  CrkL interactions have been implicated in T cell and B cell receptor, and cytokine receptor signaling.  Ref: 11399323 Mol Immunol, 2000
- Differential interaction of  **Cbl**  with  **Grb2**  and CrkL in CD2 mediated NK cell activation.  Ref: 11399323 Mol Immunol, 2000
- Here we analyzed tyrosine phosphorylation and interactions of  **Cbl**  with adapter proteins,  **Grb2**  and CrkL, in NK3.3 cells.  Ref: 11399323 Mol Immunol, 2000
- The data are consistent with the model whereby  **Grb2**  participates in EGFR internalization through the recruitment of  **Cbl**  to the receptor, thus allowing proper ubiquitylation of EGFR and or associated proteins at the plasma membrane.  Ref: 12631709 Mol Biol Cell, 2003
- The  **Grb2**  and the Crk L binding regions were mapped to the C terminus of  **Cbl**  b.  Ref: 10022120 Oncogene, 1999
- Ligand induced desensitization of the epidermal growth factor receptor EGFR is controlled by c  **Cbl** , a ubiquitin ligase that binds multiple signaling proteins, including the  **Grb2**  adaptor.  Ref: 11823423 EMBO J, 2002
- We used the U937 cell line to examine the modulation of adaptor protein interactions Shc,  **Grb2** , and  **Cbl**  after high affinity IgG receptor FcgammaRI cross linking, leading to the formation of the  **Grb2**  Sos complex, the activation of Ras, and the regulation of the respiratory burst.  Ref: 10570290 J Immunol, 1999
- Phosphorylation of LAT creates binding sites for the Src homology 2 SH2 domains of other proteins, including PLC gamma1,  **Grb2** , Gads, Grap, 3BP2, and Shb, and indirectly binds SOS, c  **Cbl** , Vav, SLP 76, and Itk.  Ref: 11752630 Sci STKE, 2000
- Kinetic analysis reveals that the tyrosine phosphorylation of  **Cbl**  is coincident with its plasma membrane translocation and association with the activated tyrosine phosphorylated CSF 1 R, p85,  **Grb2** , and tyrosine phosphorylated p58Shc.  Ref: 10025673 J Cell Biochem, 1999
- **Cbl**  is a cytosolic protein that is rapidly tyrosine phosphorylated in response to Fc receptor activation and binds to the adaptor proteins  **Grb2** , CrkL, and Nck.  Ref: 10204582 J Leukoc Biol, 1999
- Interestingly, TCDD decreases the tyrosine phosphorylation level of  **Cbl** , although  **Cbl**  recruits more  **Grb2**  and Shc proteins by TCDD.  Ref: 9918770 Biochem Biophys Res Commun, 1998
- Overexpression of c  **Cbl**  mutants that are capable of  **Grb2**  binding but defective in linker RING finger domain function severely inhibited epidermal growth factor receptor internalization.  Ref: 12839496 Traffic, 2003
- **Grb2**  SH3 domains formed an association with the proline rich sequences of Sos and  **Cbl**  in both resting and activated platelets, since the peptidimer abolished these associations.  Ref: 11964172 Biochem J, 2002

**Non-interactive relationship** (e.g. studied together, co-existance, homology, etc.)

- **Grb2**  and  **Cbl**  binding to SLP 76 were inducible after Fc gammaRI stimulation of the macrophages.  Ref: 10204582 J Leukoc Biol, 1999
- The signaling proteins, Vav,  **Grb2** ,  **Cbl**  and Gab2, and the endocytic proteins, AP2 and clathrin, all map to the primary domains, while the p85 regulatory subunit of phosphatidylinositol 3 PI 3 kinase maps to both domains.  Ref: 12217393 Mol Immunol, 2002
- Although the precise roles of interactions of  **Cbl**  with  **Grb2**  and CrkL in NK cell activation remains to be elucidated, their tyrosine phosphorylation, in addition to the multiple protein interactions described here, strongly suggest that interactions of  **Cbl**  with  **Grb2**  and CrkL may play pivotal roles in CD2 mediated NK cell activation.  Ref: 11399323 Mol Immunol, 2000
- Co immunoprecipitations showed EGFR in complex with both Shc,  **Grb2**  and  **Cbl** .  Ref: 11370743 Eur J Cell Biol, 2001
- The present study is designed to clarify whether TCDD modulates the molecular interactions of Shc,  **Cbl** ,  **Grb2** , and Sos in primary rat hepatocytes.  Ref: 9918770 Biochem Biophys Res Commun, 1998
- Complexes formed by Sos and  **Cbl**  are largely independent and membrane complexes of  **Cbl**  with other tyrosine phosphorylated proteins, p85 and  **Grb2**  also contain CSF 1 R.  Ref: 10025673 J Cell Biochem, 1999
- A small pool of CSF 1R formed a multimeric complex with phosphatidylinositol 3 kinase PI 3 kinase, SHP 1,  **Grb2** , Shc, c Src,  **Cbl** , and a significant number of tyrosine phosphorylated proteins in CSF 1 stimulated cells.  Ref: 10330148 Mol Cell Biol, 1999
- Furthermore, tyrosine phosphorylation of  **Cbl**  may not be critical for interaction of the protein with  **Grb2**  and Shc in the TCDD signaling pathway in primary rat hepatocytes.  Ref: 9918770 Biochem Biophys Res Commun, 1998
- These data indicate that the stimulation of FcalphaR results in the modulation of adaptor complexes containing tyrosine phosphorylated  **Cbl** , Shc, SHIP,  **Grb2** , and Crkl.  Ref: 10477741 Blood, 1999
- However, costimulation of IL 2 with CD2 crosslinking remarkably enhanced associations of  **Grb2**  Shc and CrkL  **Cbl** , compared to IL 2 stimulation or CD2 crosslinking alone.  Ref: 11792427 Mol Immunol, 2002
- Utilizing unique phospho specific antibodies generated against various tyrosines of c Met, we show that Y1003 binding site for c  **Cbl**  and a negative regulatory site, Y1313 binding site for PI3K, Y1230 Y1234 Y1235 autophosphorylation site, Y1349 binding site for  **Grb2** , Y1365 important in cell morphogenesis are phosphorylated in response to HGF 40 ng ml, 7.5 min in H69 cells.  Ref: 12611639 J Cell Mol Med< MedlineTA>J Cell Mol Med, 2002
- It was found in complexes with many signaling proteins, including phosphoinositol PtdIns 3 kinase EC 2.7.1.137,  **Cbl** ,  **GRB2** , p130Cas and Crk.  Ref: 12135478 Eur J Biochem, 2002
- EphA activation also induced  **Cbl**  association with Crk L and Crk II adapters, but not the related  **Grb2**  protein.  Ref: 12794130 J Immunol, 2003
- Furthermore, the EGFR mutant lacking  **Grb2**  binding sites did not efficiently recruit c  **Cbl**  and was not polyubiquitinated.  Ref: 12631709 Mol Biol Cell, 2003
- We show that a substantial fraction of tyrosine phosphorylated epidermal growth factor receptor and Shc,  **Grb2**  and  **Cbl**  after internalization relocates from early endosomes to compartments which are negative for the early endosomes, recycling vesicle markers EEA1 and transferrin in EGF stimulated cells.  Ref: 11370743 Eur J Cell Biol, 2001
- Activation of muscarinic receptors with carbachol was found to inhibit EGF induced signaling, including tyrosine phosphorylation of the adaptor protein  **Cbl**  and of the EGF receptor, and complex formation between Shc proteins and the EGF receptor and  **Grb2** .  Ref: 10049786 Biochem Biophys Res Commun, 1999
- FRS2 alpha attenuates FGF receptor signaling by  **Grb2**  mediated recruitment of the ubiquitin ligase  **Cbl** .  Ref: 11997436 Proc Natl Acad Sci U S A, 2002
- About 50% of the T cells from young mice that formed conjugates with peptide pulsed APC were found to form complexes, at the site of binding to the APC, containing CD3epsilon, linker for activation of T cells LAT, and Zap 70 in a central area and c  **Cbl** , p95 vav, Grb 2  [ **GRB2** ] , PLC gamma, Fyn, and Lck distributed more uniformly across the interface area.  Ref: 11207267 J Immunol, 2001
- Our results provide the first evidence that FcalphaR mediated signals modulate a multimolecular adaptor protein complex containing  **Grb2** , Shc, SHIP, CrkL,  **Cbl** , and SLP 76.  Ref: 10477741 Blood, 1999
- The transmembrane adapter LAT coprecipitates with SLP 76 and PLCgamma2, as well as with a number of other adapter proteins, some of which have not been previously described in platelets, including  **Cbl** ,  **Grb2** , Gads, and SKAP HOM.  Ref: 10942756 J Biol Chem, 2000
- The underlying mechanism seems to involve recruitment of a  **Grb2**  c  **Cbl**  complex to  **Grb2**  specific docking sites of EGFR, and concurrent acceleration of receptor ubiquitylation and desensitization.  Ref: 11823423 EMBO J, 2002
- In contrast to constitutive  **Cbl**  association with  **Grb2** , tyrosine phosphorylated  **Cbl**  interacts with CrkL via its SH2 domain only after CD2 stimulation.  Ref: 11399323 Mol Immunol, 2000
- Our results shed light on the mechanism of receptor desensitization mediated by c  **Cbl**  and its binding partner  **Grb2** .Oncogene 2003 2 669 3679.  Ref: 12802274 Oncogene, 2003
- The membrane translocation and the pattern of association of Sos with the CSF 1R, p85,  **Grb2** , and p58Shc resemble those of  **Cbl**  but Sos is not tyrosine phosphorylated, nor multiubiquitinated and the coprecipitation of these proteins, other than  **Grb2** , with Sos is much less.  Ref: 10025673 J Cell Biochem, 1999
- Association of Shc,  **Cbl** ,  **Grb2** , and Sos following treatment with, tetrachlorodibenzo p dioxin in primary rat hepatocytes.  Ref: 9918770 Biochem Biophys Res Commun, 1998
- The PER mutant with all five autophosphorylation sites mutated to phenylalanine 5YF was equivalently capable of interacting with several important signaling molecules, including Shc,  **Grb2** , Gab1, phospholipase Cgamma, and  **Cbl** .  Ref: 12615082 Int J Dev Neurosci, 2003
- There is an activation induced association of tyrosine phosphorylated  **Cbl**  with  **Grb2**  and CrkL, respectively, but not CrkII.  Ref: 11263968 Biochem Biophys Res Commun, 2001
- In the membrane fraction of cells stimulated at 4 degrees C, the association of p58Shc and  **Grb2**  with  **Cbl**  is stable.  Ref: 10025673 J Cell Biochem, 1999
- Kinetic experiments confirm that  **Cbl**   **Grb2**  is relatively stable.  Ref: 10570290 J Immunol, 1999
- NTAL becomes rapidly tyrosine phosphorylated upon cross linking of the B cell receptor BCR or of high affinity Fcgamma and Fc varepsilon receptors of myeloid cells and then associates with the cytoplasmic signaling molecules  **Grb2** , Sos1, Gab1, and c  **Cbl** .  Ref: 12486104 J Exp Med, 2002
